# Rapid NGS Analysis on Google Cloud Platform: Performance Benchmark and User Tutorial

**DOI:** 10.1111/cts.70416

**Published:** 2025-11-18

**Authors:** Eugenio Franzoso, Mariangela Santorsola, Francesco Lescai

**Affiliations:** ^1^ Department of Biology and Biotechnology “L. Spallanzani” University of Pavia Pavia Italy

## Abstract

Next‐Generation Sequencing (NGS) is being increasingly adopted in clinical settings as a tool to increase diagnostic yield in genetically determined pathologies. However, for patients in critical conditions the time to results of data analysis is crucial for a rapid diagnosis and response. Sentieon DNASeq and Clara Parabricks Germline are two widely used pipelines for ultra‐rapid NGS analysis, but their high computational demands often exceed the resources available in many healthcare facilities. Cloud platforms, like Google Cloud Platform (GCP), offer scalable solutions to address these limitations. Yet, setting up these pipelines in a cloud environment can be complex. This work provides a benchmark of the two solutions, and offers a comprehensive tutorial aimed at easing their implementation on GCP by healthcare bioinformaticians. Additionally, it presents valuable cost guidance to healthcare managers who consider implementing cloud‐based NGS processing. Using five publicly available exome (WES) and five genome (WGS) samples, we benchmarked both pipelines on GCP in terms of runtime, cost, and resource utilization. Our results show that Sentieon and Parabricks perform comparably. Both pipelines are viable options for rapid, cloud‐based NGS analysis, enabling healthcare providers to access advanced genomic tools without the need for extensive local infrastructure.

## 1 Introduction

There are several scenarios where a very rapid diagnosis of a patient can make the difference [1, 2]. This is particularly relevant, but not limited, to the case of critically ill pediatric patients who are in intensive care units (PICU and NICU) [3, 4]. In these contexts, Next‐Generation Sequencing (NGS) has emerged as a crucial tool in the diagnostic process, because it has dramatically increased the diagnostic yield compared to traditional diagnostics [5, 6]. Several recent studies have demonstrated how rapid whole‐exome or genome sequencing (WES/WGS) can influence diagnosis and clinical management within days [2, 7, 8, 9, 10, 11, 12]. For example, Ball et al. [8] diagnosed mitochondrial disorders in critically unwell infants using ultra‐rapid sequencing, enabling targeted interventions and guiding urgent medical decisions. Kingsmore et al. [2] reviewed multiple ICU cases where rapid WGS provided a diagnosis in under 48 h, resulting in modified treatment plans, initiation of palliative care, or withdrawal of unnecessary interventions. Similar outcomes were reported by Auber et al. [9] and Marouane et al. [13], further reinforcing that while not common across all patients, in specific, high‐risk scenarios, the ability to obtain a genetic diagnosis within hours to a few days can be life‐saving and cost‐effective.

However, while introducing indisputable advantages, NGS increases the burden in data analysis and interpretation leading to diagnosis [14, 15]. Therefore, while other steps of the clinical workflow have little margins for speed‐ups, innovations in bioinformatics have the potential to dramatically impact the time‐to‐diagnosis in these scenarios. Amongst the available tools on the market for ultra‐rapid NGS data processing are Sentieon [16] and Clara Parabricks [17]. The first one accelerates the analysis by differently exploiting Central Processing Units (CPUs), while the second one accelerates the workflow by using Graphical Processing Units (GPUs). By accelerating the bioinformatic analysis, these tools allow clinicians to make a faster diagnosis, while also leading to substantial cost savings for healthcare providers [18, 19]. Despite these improvements, a major hurdle remains: the requirement of substantial computational resources, such as large multicore servers or GPU cards, often unavailable in many hospital settings. Generally, a small/medium on‐premises HPC scratch storage setup (~1 PB targeting ~10 GB/s throughput) costs around US $150,000 initially, with ~30% annual maintenance (~US 45,000/year) [20]. Entry‐level HPC clusters range from US $50,000 to US $250,000, depending on size, plus ongoing operating expenses such as power, cooling, and staffing [21]. Adopting cloud‐based solutions allows healthcare providers significant cost savings by eliminating the need to maintain expensive on‐premises hardware and infrastructure, and ongoing maintenance. Instead of incurring capital expenditures, healthcare providers can use operational expenditure, paying on‐demand solely for the resources they use. Therefore, a cloud‐first strategy appears to be a practical and feasible solution in many settings. Cloud computing simplifies the deployment of these advanced software to institutions lacking the necessary infrastructure, while maintaining compliance with regulatory requirements [22]. Besides providing a flexible, maintained and backed‐up computing solution, the adoption of a cloud solution makes costs predictable and proportional to the actual demand.

Cloud computing and bioinformatics are already closely integrated, providing secure and flexible solutions for storage and analysis of large‐scale genomic, proteomic, and metabolomic data, which are often considered pivotal for the advancement of personalized medicine [22]. Currently, cloud computing is a rapidly growing alternative to local infrastructures, offering scalable and efficient computational environments for high‐throughput NGS analyses across a variety of applications [23, 24, 25, 26, 27]. Several platforms, such as the Globus Genomics [23], Genomics Virtual Laboratory [24], NGScloud2 [25], demonstrate the growing adoption of cloud‐based solutions for large genomic datasets.

On these grounds, we have benchmarked Sentieon DNASeq and Clara Parabricks Germline to measure their performance in terms of speed and costs on the Google Cloud Platform (GCP). We focused our comparison on key parameters such as sample processing time, cost per sample, CPU and memory usage as well as ease of implementation. The goal of this benchmark is to provide healthcare managers and clinical bioinformaticians with a high‐level estimate of resources and a step‐by‐step guideline to facilitate the implementation of ultra‐rapid NGS solutions on GCP cloud, either as a standalone strategy or to complement existing infrastructures already burdened by intensive workloads, particularly for routine or time‐sensitive analyses.

### 1.1 Samples and Data Availability

This benchmarking was designed to illustrate a ready‐to‐use workflow for ultra‐rapid NGS analysis in a hypothetical healthcare setting, rather than to compare performance across diseases or therapeutic areas. To ensure full reproducibility, we selected openly available FASTQ files representative of typical WES and WGS datasets. Specifically, we assessed the performance of Sentieon DNASeq and Clara Parabricks Germline for ultra‐rapid NGS data processing of human data, using five whole‐exome (WES) and five whole genome (WGS) samples from the Sequence Read Archive (SRA).

The WES data we selected belong to a study focused on patients with a syndromic condition characterized by lymphoproliferation, immunodeficiency, and hemophagocytic lymphohistiocytosis (HLH) like phenotypes [28]. The genomic DNA extracted from these patients underwent exome enrichment using the Twist Core Exome capture system. Sequencing was performed on an Illumina NextSeq 500 platform, with a paired‐end 75 base pairs (bp) read length.

The WGS data belong to Illumina's Polaris project, aimed at developing publicly available resources for population genomics analyses [29]. The samples were obtained from sequencing on an Illumina HiSeqX sequencer using a 150 bp read length.

Detailed information regarding the individual sample identifiers and corresponding data can be found in Table S1.

### 1.2 Design of the Benchmark

We processed the ten samples from the raw FASTQ files to VCF using two state‐of‐the‐art, ultra‐rapid germline variant calling pipelines: Sentieon's DNASeq v202308 [30] and Parabricks Germline v4.0.1–1 [31]. To ensure a standardized comparison, both pipelines were launched with their default parameters and execution steps, including alignment, marking duplicates, base recalibration and variant calling.

**FIGURE 1:**
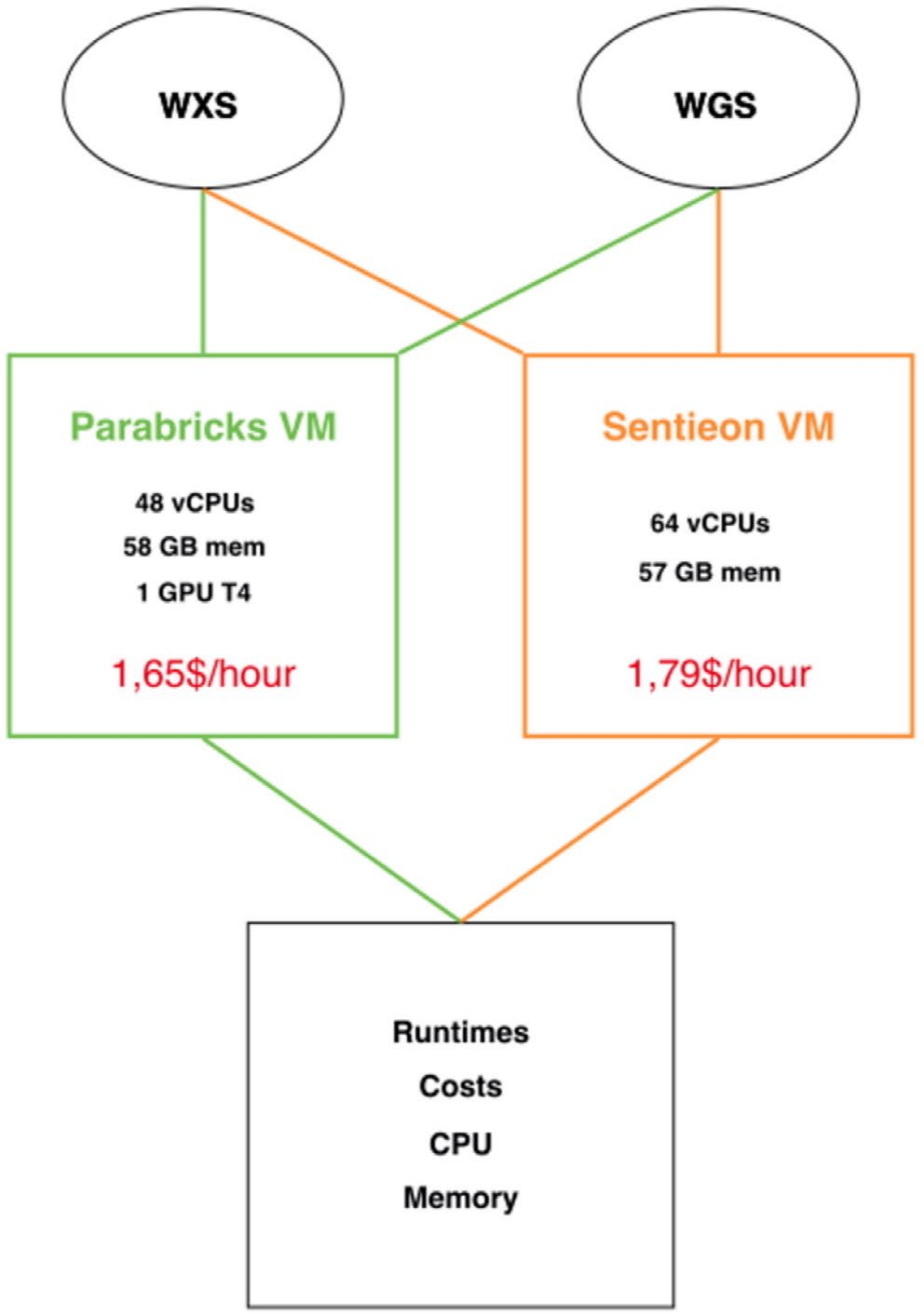
Whole‐exome (WES) and whole‐genome (WGS) samples were processed on two distinct VMs by Sentieon and Parabricks, each chosen based on a comparable baseline cost per hour. The benchmarking parameters are indicated in the square at the bottom of the image.

To accommodate the distinct hardware requirements of each pipeline, we utilized GCP to set up two dedicated virtual machines (VMs), each one tailored for either of the pipelines. Figure 1 illustrates the overall design of our benchmarking analysis, focused on evaluating and comparing the performance of each pipeline in terms of runtime, overall cost, and resource allocation (CPU and memory usage).

### 1.3 Cloud Deployment Design and Implementation

To support healthcare facilities in implementing ultra‐rapid NGS workflows, we provide a comprehensive step‐by‐step tutorial for deploying two industry‐standard pipelines, Sentieon and NVIDIA Clara Parabricks, on the GCP. This work integrates benchmarking design with practical implementation, offering a hands‐on and executable guide.

To choose the VMs for this benchmark, we aimed at comparable baseline costs per hour, while meeting each software's requirements. This cost‐driven approach was meant to evaluate the performances the two pipelines would achieve in a similar cost‐constrained scenario.

The VM for Sentieon DNASeq was designed with 64 vCPUs and 57GB of memory, aligning with the tool's CPU‐based processing requirements: this VM had a baseline cost of $1.79/h. The VM for Clara Parabricks Germline was instead configured with 48vCPUs, 58 GB of memory, and 1 T4 NVIDIA GPU: this VM had a baseline cost of 1.65$/h.

What follows is a practical tutorial for deploying and executing both pipelines in these configurations.

### 1.4 Step‐By‐Step Tutorial for Deploying Ultra‐Rapid NGS Pipelines on GCP

This tutorial is intended for users with varying levels of technical expertise, but dedicated to data analysis, and requires only basic familiarity with the bash shell and access to a GCP project with billing enabled.

**1. Prerequisites and user requirements**

To follow this section, users will need:

- An GCP account with billing enabled
- Basic familiarity with the bash shell
- A valid license for running Sentieon (not required for Parabricks)

**2. Sentieon DNASeq setup on GCP**

This section outlines the complete process for configuring and running Sentieon DNASeq on a CPU‐based VM.

**2.1. VM Configuration**

1. Once the license has been purchased, you will receive the links for downloading it together with the software. Use the links you have received to download and save both the license file and the software folder on your machine. These will be transferred later to the VM using a Secure Copy Protocol (SCP).
2. Go to console.cloud.google.com/compute/instances.
3. Click “CREATE INSTANCE”. You will be redirected to a new page where you can choose how to configure your instance.
4. Enter:
  - Name for the VM
  - Region and zone depending on your location (e.g., europe‐west4 and europe‐west4‐a, respectively).
5. Under “MANAGE TAGS AND LABELS” add:
  - Key: “machine”
  - Value: “sentieon”
6. In the “Machine Configuration” section:
  - Select the N1 series.
  - Under “Machine Type”, choose n1‐highcpu‐64 (64vCPU, 32 core, 57.6 GB memory).
7. In the Boot Disk section:
  - Click “CHANGE”
  - Chose CentOS7 as the public image
  - Leave the boot disk type untouched
  - Set disk size (we used 500 GB)
8. Check the “Install Ops Agent for monitoring and logging” under the Observability, Ops Agent section.
9. Click “CREATE” at the bottom of the page, and wait for the VM to instantiate. This may take a while.
10. Go back to console.cloud.google.com/compute/instances. When the VM is ready, click “SSH” to open a shell session.

**2.2 Environment setup**

1. Install the required software:

sudo yum install git screen bzip2 libxml2-dev wget

2 To transfer files from a local infrastructure to GCP, a ssh key must be added to

~/.ssh/authorized_keys file



on the VM.

On your local machine, create an SSH key for file transfer:ssh-keygen -t rsa -f ~/.ssh/gcloud_key -C/local_username -b 2048


This will create a public key in your local ~/.ssh folder, named gcloud_key.pub. Use the cat command to visualize the key and copy it entirely:cat ~/.ssh/gcloud_key.pub

3 On the VM, add the public key:

vim ~/.ssh/authorized_keys



Paste the key you have just copied on your local machine in the file, making sure that the format is the same as the other keys that have been added automatically during the instantiation of the VM by Google.

Modify the last part of the key (local_username), where your username is specified.

Save with: wq.

Example of the SSH key format required:ssh-rsa A​AeA​B3N​zaC​1yc​2EA​AAA​DAQ​ABA​AAB​AQC​7NX​4iK​XDX​Ysf​FEa​c9Q​SQS​I6x​z5H​69e​tM+​GIK​hQj​x5M​hby​Zca​R/f​WPx​hU6​DK9​vu8​PIa​ZOM​N7i​lh6​k/m​llC​1LZ​nLn​ibW​w5z​8Vr​w1S​PPa​DF5​6SH​1hc​Wa1​+1p​gPG​+89​v1j​zKl​8rJ​Lqf​OlX​kIS​SYG​jWX​Y/y​vo6​N/M​2pD​q/X​U7u​h3t​xFh​Gca​m9B​cUx​nsq​Jhn​PCs​Hv/​+P9​A1e​owO​Ikc​Faz​J+y​3ct​g9M​hhC​/jb​lGV​QLE​wl6​nCF​sZp​sVZ​yFw​aB8​kKV95wnD9SvGHHPYWphIJPASKtr+qFSGt75H4yrjRgkCRYH02JQgpLfW1jo/sPs0c4iKaVhO1oi8uaqJL8tTFJtKGOHvFHz local_username {“userName”:“local_username”,“expireOn”:“YYYY-MM-00T09:58:35+0000”}


**2.3 File transfer and directory setup**

On the VM home create two new folders: one for the Human genome references and one for the sample.mkdir -p referencesmkdir -p sample


From your local machine, transfer files using scp:scp -r -i/home/user/.ssh/gcloud_key/home/path/to/reference/files gcloud_username@external_ip_address:/home/gcloud_username/references/


Repeat the command to transfer samples (to/sample folder), software license and software folders to the VM/home folder.

**2.4 Workflow execution**

1. Unzip the software folder on VM:

tar xvzf sentieon-genomics-202308.02.tar.gz

2 Move the software license in a directory named LICENSE_DIR. Type the following commands:

mv LICENSE_FILE.lic LICENSE_DIR/.export SENTIEON_LICENSE=LICENSE_DIR/LICENSE_FILE.lic

3 Obtain the Sentieon official workflow scripts at https://github.com/Sentieon/sentieon‐scripts/blob/master/example_pipelines/germline/DNAseq. Our customized scripts are available at: https://github.com/lescailab/genomics‐benchmarks/tree/main/sentieon/sentieon_gcp/scripts.
4 Download the script you need, edit it and transfer it to the VM with the following command:

scp -r -i/home/user/.ssh/gcloud_key/home/path/to/script/file gcloud_username@external_ip_address:/home/gcloud_username/.

5 Start a screen session in your VM terminal. Screen is a terminal multiplexer that allows you to open virtual terminals inside of your session. This means that even if your connection with the VM was interrupted, the processes running in Screen would continue to run. If you want to detach from the screen session, just press the keys ctrl+a+d.
6 Run the Sentieon DNASeq workflow:

bash <wes-interval.sh><wgs.sh>



**3. Clara Parabricks Germline setup on GCP**

This section guides users through deploying the GPU‐accelerated Clara Parabricks Germline pipeline on a custom‐configured VM in GCP.

**3.1. VM Configuration**

1. Go to console.cloud.google.com/compute/instances.
2. Click “CREATE INSTANCE”.
3. Enter:
  - Name for the VM
  - Region and zone depending on your location (e.g., europe‐west4 and europe‐west4‐a as region and zone).
4. Under “MANAGE TAGS AND LABELS” add:
  - Key: “machine”
  - Value: “sentieon”
5. In the “Machine Configuration” section:
  - Select the GPUs series.
  - Choose 1 NVIDIA T4.
  - Under “Machine Type”, click “CUSTOM”, then set:
    - 48 vCPU cores
    - 58 GB memory
6. Under the Boot Disk section:
  - Click “CHANGE”
  - Select Debian Debian GNU/Linux 10 (buster) as base image.
  - Leave the boot disk type untouched
  - Set the size disk (we used600 GB)
7. **DO NOT** check the “Install Ops Agent for monitoring and logging” under the Observability, Ops Agent section. We have experienced that this might cause an error once the VM is up and running. It will be installed later.
8. Click “CREATE” at the bottom of the page and wait for the VM instantiation. This may take a while.
9. Go back to console.cloud.google.com/compute/instances, once the VM appears, click “SSH” to connect to it.

**3.2 Environment setup**

1. Install the Ops Agent. From your/home folder:

curl -sSO https://dl.google.com/cloudagents/add‐google‐cloud‐ops‐agent‐repo.shsudo bash add-google-cloud-ops-agent-repo.sh​--also-install

2 Install NVIDIA GPU drivers:

# Ensure Python3 is installed on the systempython3--version# Download the startup scriptcurl https://raw.githubusercontent.com/GoogleCloudPlatform/compute‐gpu‐installation/main/linux/startup_script.sh‐‐outputstartup_script.sh# Download the installation scriptcurl https://raw.githubusercontent.com/GoogleCloudPlatform/compute‐gpu‐installation/main/linux/install_gpu_driver.py‐‐outputinstall_gpu_driver.py# Launch the startup scriptsudo bash startup_script.sh# Check for drivers' installationsudo nvidia-smi



**3.3. Docker and Parabricks Setup**

1. Since Parabricks can be downloaded as a container from the web, you need to install Docker on the VM. Type the following commands:

# Delete the outdated packagessudo apt-get purge docker lxc-docker docker-engine docker.io# Update the default repositorysudo apt-get update# Download the following dependenciessudo apt-get install apt-transport-https ca-certificates curl gnupg2 software-properties-common# Download Docker's official GPG key to verify the integrity of packages before installingcurl -fsSL https://download.docker.com/linux/debian/gpg|sudoapt‐keyadd# Add the Docker repository to your system repositorysudo add-apt-repository “deb [arch=amd64] https://download.docker.com/linux/debianbusterstable”# Update the apt repositorysudo apt-get update# Install Docker Engine – Community (the latest version of Docker) and containerdsudo apt-get install docker-ce docker-ce-cli containerd.io# The service will start automatically after the installation. Check the statussudo systemctl status docker# Check Docker versiondocker--version 

2 Download the Clara Parabricks container using Docker:

docker pull nvcr.io/nvidia/clara/clara-parabricks:4.0.0-1

3 Install the NVIDIA Container Toolkit:

# Configure the repositorycurl -fsSL https://nvidia.github.io/libnvidia‐container/gpgkey|sudogpg‐‐dearmor‐o/usr/share/keyrings/nvidia‐container‐toolkit‐keyring.gpg&& curl -s -L https://nvidia.github.io/libnvidia‐container/stable/deb/nvidia‐container‐toolkit.listsed’s#deb https://#deb[signed-by=/usr/share/keyrings/nvidia-container-toolkit-keyring.gpg] https://#g|\sudo tee/etc/apt/sources.list.d/nvidia-container-toolkit.list\&&\csudo apt-get update# Install the NVIDIA Container Toolkit packagessudo apt-get install -y nvidia-container-toolkit

4 Install screen for session management:

sudo apt-get install screen



**3.4. File transfer and setup**

The steps for copying reference and sample files to the VM are identical to those in the Sentieon setup. Use scp with your SSH key to transfer:

1. Reference genome files
2. Sample FASTQ files

Place them in appropriate folders (/workdir/references/workdir/sample) on the VM.

**Important! In the case of Parabricks there is no need for a license and you are going to use the container downloaded in step 11 to run the software. You only need your sample and reference files on the VM**.

**3.5. Workflow Execution**

Once the reference and sample files are transferred on your VM, launch the Clara Parabricks Germline workflow within a screen session:sudo docker run\--gpus all \--rm \--volume $(pwd):/workdir \--volume $(pwd):/outputdir \nvcr.io/nvidia/clara/clara-parabricks:4.0.0-1 \pbrun germline \--ref/workdir/references/Homo_sapiens_assembly38.fasta \--in-fq/workdir/sample/$(basename “$fwd”)/workdir/sample/$(basename “$rev”) \--knownSites/workdir/references/Homo_sapiens_assembly38.known_indels.vcf.gz \--out-bam/outputdir/“${sample_name}”_markdup.bam \--out-variants/outputdir/“${sample_name}”.vcf \--out-recal-file/outputdir/recal.txt \--tmp-dir/outputdir/tmp


The script we used for our benchmark is available here: https://github.com/lescailab/genomics‐benchmarks/blob/main/parabricks/parabricks_gcp/no_loop_runs/launch_parabricks.sh.

### 1.5 Data Collection and Analysis

To gather performance metrics, we employed the Ops Agent (https://cloud.google.com/monitoring/agent/ops‐agent) on each instance. The GCP monitoring dashboard was used to collect all the metrics, including the computation start and end, CPU and memory usage.

All the plots have been generated using R version 4.3.0 (2023‐04‐21) inside the RStudio Integrated Development Environment (IDE) (version 2023.06.0+421). The R package tidyverse (version 2.0.0) was used for data manipulation and plotting (https://www.tidyverse.org/).

### 1.6 Software Runtimes

**FIGURE 2:**
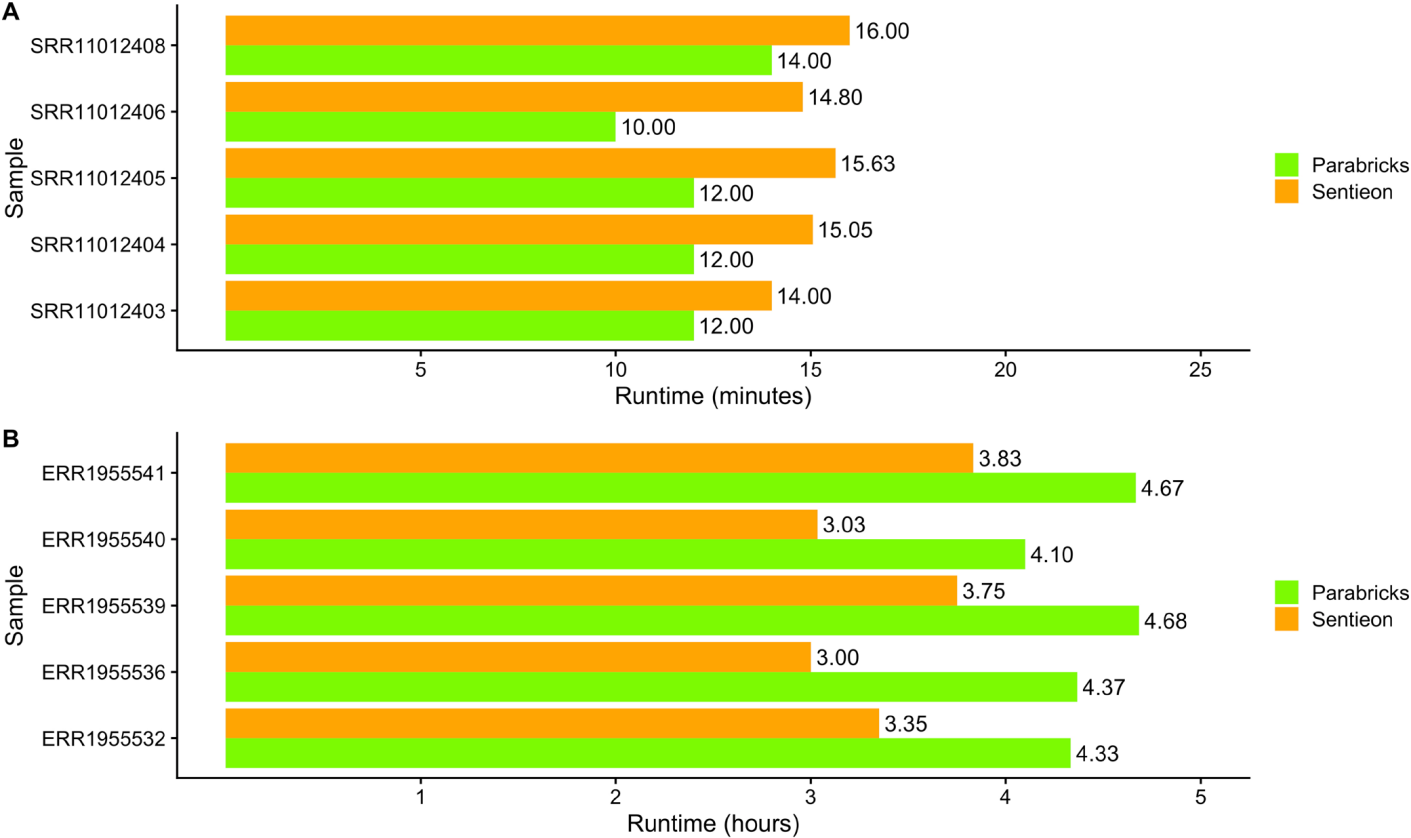
(A) Runtime analysis (in minutes) of the whole‐exome samples (WES). (B) Runtime analysis (in hours) of the whole‐genome samples (WGS).

The analysis of the WES runtimes (Figure 2A) showed that Parabricks' Germline completed the variant calling process in approximately 10–14 min. Sentieon's DNASeq displayed a similar performance, with analysis duration ranging from 14 to 16 min.

As far as the analysis of genome samples, we observed a more significant variation in processing times (Figure 2B). The elapsed time for Parabricks analysis ranged between 4.1 and 4.7 h, while Sentieon analysis lasted between 3 and 3.8 h.

### 1.7 Costs

**FIGURE 3:**
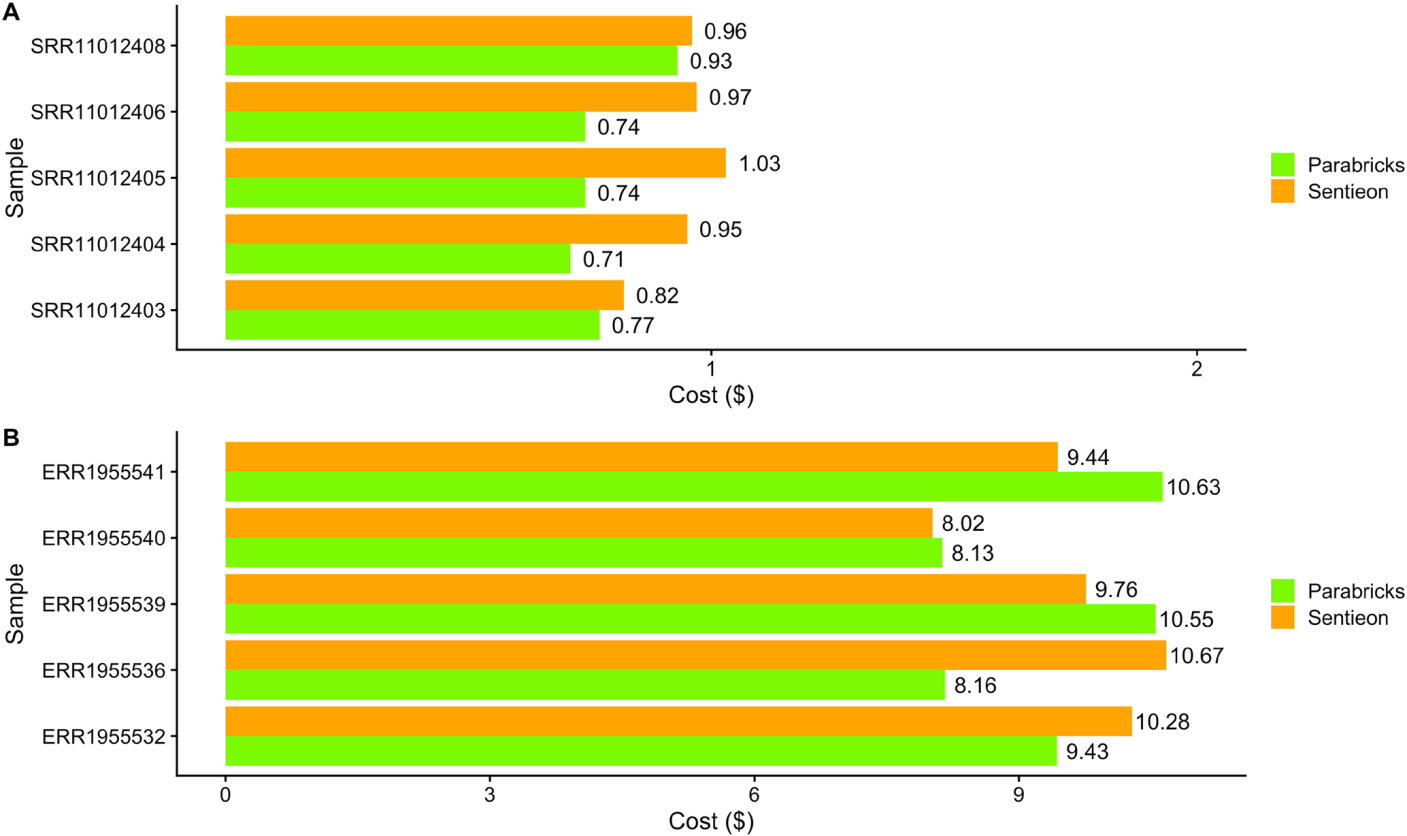
(A) Cost in USD ($) for the processing of each exome sample. (B) Cost in USD ($) for the processing of each genome sample.

The cost for processing the exome samples with Parabricks Germline ranged between 0.71$ and 0.93$. Using Sentieon the costs of the analysis ranged between 0.82$ and 1.03$ (Figure 3A).

As far as genome analyses are concerned (Figure 3B), the cost of running Parabricks ranged from 8.13$ to 10.63$ per sample while Sentieon's DNASeq cost between 8.02$ and 10.67$.

Overall, Parabricks Germline incurred lower costs on two out of five WGS datasets (ERR1955532 and ERR1955536).

Given the fundamental differences in the way Sentieon and Parabricks exploit the hardware, and in turn how this impacts these measurements, we also profiled CPU and memory to gather a more complete picture of the overall performance.

### 1.8 CPU and RAM Usage

**FIGURE 4:**
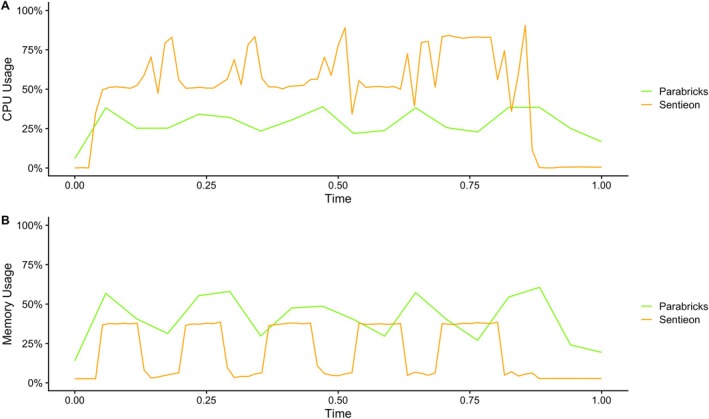
(A) CPU usage across the five WES analyses with Sentieon and Parabricks. (B) Memory usage across the five WES analyses with Sentieon and Parabricks. The *x*‐axis represents normalized runtime, where 0.0 corresponds to the start of the workflow and 1.0 corresponds to the end of the workflow.

**FIGURE 5:**
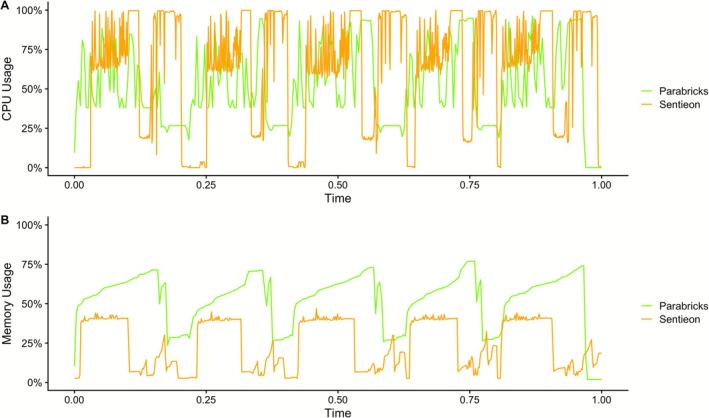
(A) CPU usage across the five WGS analyses with Sentieon and Parabricks. (B) Memory usage across the five WGS analyses with Sentieon and Parabricks. The *x*‐axis represents normalized runtime, where 0.0 corresponds to the start of the workflow and 1.0 corresponds to the end of the workflow.

We profiled in detail both CPU and memory usage throughout the analyses carried out by Sentieon and Parabricks, across all WES (Figure 4) and WGS (Figure 5) samples. The analysis of the measurements showed, as expected, that Sentieon is quite greedy in using the available CPUs but not as greedy in using the available memory. On the other hand, Parabricks, despite being accelerated on the GPU which has its own dedicated memory, showed a higher and constant usage of the mainboard memory. At the same time, during the genome samples analysis Parabricks also showed a significant usage of the available (16) CPUs.

## 2 Discussion

The benchmark presented in this study provides a snapshot of runtime, costs and technical expertise required when performing ultra‐rapid NGS data analyses on GCP, with a focus on clinical applicability.

The decline in the cost of sequencing, along with its high diagnostic yield, has opened new avenues in clinical settings, especially when implemented as a first‐line diagnostic test [5, 6]. However, to fully leverage these benefits, it is essential to pair sequencing with accurate and rapid bioinformatic approaches, accelerating genetic and physician consultations, and then the route to diagnosis.

By comparing two leading tools, Sentieon and Clara Parabricks, under matched cost constraints, our study provides healthcare institutions with practical insights into performance, scalability, and feasibility. Beyond technical benchmarking, we focus on real‐world implementation challenges and aim to support healthcare institutions at different stages of digital maturity, including those with limited bioinformatics expertise, but primarily dedicated to data analysis, or infrastructure.

Nowadays cloud‐based solutions enable healthcare providers to overcome the limitations of on‐site hardware, by reducing the time and costs associated with its maintenance and offering scalable and flexible configurations tailored to specific needs [22]. Additionally, cloud providers manage system updates, security, and maintenance, which reduces the need for in‐house IT staff and lowers overall operational costs [32]. For example, benchmarking studies using Amazon Web Services (AWS) for molecular simulations (e.g., GROMACS), showed that cloud‐based setups, accounting for all costs including hardware, energy, and personnel, were found to be as cost‐efficient or cheaper than a typical on‐premises cluster, completing workflows 2 days instead of weeks [33]. An older enterprise case study (non‐healthcare) showed migrating to EC2 could cut infrastructure costs by ~37% over 5 years and reduce support calls by ~21% [34]. The costs associated with cloud computing are therefore predictable and directly proportional to the hardware and services chosen, facilitating straightforward budget management.

Our benchmark revealed that, overall, Sentieon and Parabricks on GCP yield comparable results in terms of runtime and costs. By standardizing the hourly cost of each virtual machine (VM), we ensured a fair comparison of performance metrics across the ten samples analyzed in a similar cost‐constrained scenario.

While Parabricks performed better than Sentieon in the analysis of exome samples, Sentieon demonstrated faster runtimes when processing genome ones. This suggests that Sentieon may utilize computational resources more efficiently when handling large datasets in the configurations we tested.

The costs we observed for exome sequencing samples were in line with expectations, with lower prices corresponding to shorter analysis times for both software.

Interestingly, we did not always observe a linear correlation between runtimes and costs when comparing both software (Table S2). For instance, samples ERR1955532 and ERR1955536 were less expensive when running Parabricks, despite taking longer than Sentieon to complete.

Based on our results, both Sentieon and Parabricks are viable solutions for performing ultra‐rapid NGS on GCP, exhibiting comparable runtimes and costs. However, some important considerations must be addressed when selecting one pipeline over the other.

Firstly, the costs highlighted in our benchmark do not include the licensing fees required for running Sentieon. In contrast, Parabricks has no licensing costs, but it requires a GPU. While deploying a GPU on‐premises (HPC) would be expensive, on GCP its cost is included in the hourly price of the virtual machine.

Another important factor is the ease of configuring the virtual machines. Setting up the VM for Sentieon was straightforward, as the software exclusively relies on CPU resources. However, configuring the VM for Parabricks was more complex due to its need for GPU acceleration, which requires appropriate GPU drivers and CUDA (Compute Unified Device Architecture) libraries compatible with the selected GPU: the “Deep Learning on Linux” images on GCP did not work for us, requiring us to configure the entire machine from scratch. The detailed procedures for custom setups for both Parabricks VM and Sentieon VM on GCP are provided in the Materials and methods section of this paper. The workflow is designed to be reproducible and accessible, requiring only basic command‐line experience. The CPU usage profiles suggest important recommendations when choosing the machine to be dedicated to each of the software: when running Sentieon, as one might expect, it is important to choose a virtual machine with a sufficient number of cores, which we evaluated to be between 64 and 96; when choosing the virtual machine for Parabricks, one should obviously choose a performant GPU, but also consider choosing a sufficient amount of memory (48–64) and a minimum of 16 CPUs. The recommended number of CPUs for Parabricks seems to be an important requirement: we observed (data not shown) that providing a lower number of CPUs, when analyzing genome samples, would drastically slow down the analysis runtimes.

A recent study by Samarakoon et al. (2025) benchmarked multiple pipelines, including Sentieon and Parabricks, on AWS [35]. While their work offers detailed performance metrics, it is primarily aimed at experienced bioinformaticians and lacks a user‐oriented implementation guide. In contrast, our study provides a practical, implementation‐focused guide suitable not only for clinical institutions but also for research centres lacking adequate local infrastructure or seeking to complement it with flexible, cloud‐based resources. The tutorial supports users with varying technical backgrounds, including entry‐level bioinformaticians, through a fully reproducible, step‐by‐step workflow tailored to GCP environments. By focusing on deployment within GCP, our work addresses real‐world challenges associated with adopting cloud solutions, particularly in healthcare settings with limited local infrastructure or specialized technical personnel. Although this shift toward cloud computing brings substantial advantages, it also introduces serious challenges in protecting sensitive health data [36]. Healthcare organizations using cloud platforms must comply with stringent data protection regulations, including the Health Insurance Portability and Accountability Act (HIPAA) in the U.S. and the General Data Protection Regulation (GDPR) in the EU. These frameworks mandate high standards for safeguarding sensitive patient data (PHI). While cloud service providers may offer HIPAA and GDPR‐compliant infrastructure, the responsibility for data protection ultimately lies with the healthcare organization. Achieving compliance requires secure system configuration, continuous auditing, and close coordination between healthcare organizations and cloud service providers. Key security mechanisms, including encryption, identity and access management (IAM), and data segmentation, are critical to maintaining compliance. A concrete example of cloud‐based NGS analyses is the Computational Genomic platform developed by the INFN‐IRCCS AOU Sant'Orsola collaboration in Italy, deployed on the high‐security, ISO certified EPIC partition of INFN Cloud, with GDPR‐compliant genomics pipeline [37].

Finally, while our benchmark focuses on the deployment of ultra‐rapid variant calling workflows on the cloud, a key step that can be significantly accelerated, it is important to acknowledge that variant interpretation remains the most complex and time‐intensive phase of clinical genomics and is beyond the scope of this study. Accurate interpretation requires the integration of various data sources, including phenotype information (e.g., OMIM [38], HPO [39]), population allele frequencies (e.g., gnomAD [40]), and clinical classifications (e.g., ClinVar [41]), to annotate, filter and prioritize actionable clinically relevant variants. Currently, no bioinformatics pipeline can fully automate the interpretation process, which is why the roles of genetic counselors and clinical geneticists are essential for contextualizing findings. Effective interpretation directly impacts diagnostic accuracy, prognosis, treatment decisions, and can help prevent unnecessary testing. This highlights its clinical value, even though it is not explored in this work.

## 3 Conclusions

This benchmark provides a valuable resource for healthcare managers and clinical bioinformaticians seeking to implement ultra‐rapid NGS solutions in their institutions, overcoming the limitations of on‐site computational resources.

By providing a high‐level estimate of the runtimes, costs, CPU, and memory usage for Sentieon and Parabricks on GCP, alongside a step‐by‐step guideline for custom VM implementation in cost‐sensitive scenarios, we aimed to facilitate the widespread adoption of these solutions in clinical settings.

With the adoption of these tools, clinicians will be able to make faster diagnoses and, ultimately, improve patient care.

## Conflicts of Interest

The authors declare no conflicts of interest.

## Supporting information


**Table S1:** Size and identifiers of the publicly available samples used for 760 the benchmark.

**Table S2:** Runtime and costs for each of the samples analyzed.
